# Comparison of the immediate analgesic effect of perpendicular needling and transverse needling at SP6 in patients with primary dysmenorrhea: Study protocol for a randomized controlled trial

**DOI:** 10.1097/MD.0000000000018847

**Published:** 2020-01-17

**Authors:** Mohammad Reza Afshari Fard, Ali Mohammadi, Liang-Xiao Ma, Jie-dan Mu, Wen-Yan Yu, Yue Song, Jun-Xiang Wang, Ying-Ying Gan, Yuan Tian, Xu Qian, Tian-Yi Sun, Somayeh Iravani

**Affiliations:** School of Acupuncture-Moxibustion and Tuina, Beijing University of Chinese Medicine, Beijing, China.

**Keywords:** clinical trial, hemodynamic mechanism, perpendicular needling, primary dysmenorrhea, Sanyinjiao SP6, transverse needling, VAS

## Abstract

**Background:**

Acupuncture has been widely used to treat primary dysmenorrhea (PD) with satisfactory outcomes. Sanyinjiao (SP6) is the most commonly used acupoint for PD. Different needling techniques may influence the effect of SP6, and its underlying mechanism needs to be explored. This randomized controlled parallel trial is designed to evaluate the immediate analgesic effect and hemodynamic responses in uterine arterial blood flow of perpendicular needling and transverse needling at SP6 in patients with PD of cold-dampness stagnation pattern using color doppler ultrasonography.

**Methods:**

Forty-eight patients who meet inclusion criteria will be randomized in a ratio of 1:1 to either perpendicular needling or transverse needling groups. Every participant will receive 1 session of acupuncture treatment for 10 minutes at bilateral SP6. In the perpendicular needling group, needles will be inserted vertically 1 to 1.2 cun and will be manipulated to achieve needling sensation. In transverse needling group, the needles will be inserted transversely 1 to 1.2 cun toward the abdomen without any manipulation to avoid needling sensation. Color doppler ultrasonography will be performed before, during, and after needling. The primary outcome measure is visual analog scale for pain. The secondary outcome measures include the uterine artery blood flow changes by measuring pulsatility index, resistance index values, and ratio of systolic peak and diastolic peak, the Hamilton anxiety scale, blood pressure, and heart rate. Adverse events in both groups also will be recorded.

**Discussion:**

This trial will be the first study protocol designed to explore the influence of needling techniques on the analgesia effect of solo acupoint and its hemodynamic responses for PD. It will promote more widespread awareness of the benefits of using suitable needling techniques in acupuncture clinical setting and provide a further explanation of the underlying hemodynamic mechanism.

**Trial registration:**

This study protocol was registered at the Chinese clinical trial registry (ChiCTR1900026051).

## 1 Introduction

Dysmenorrhea is defined with abdominal pain, which usually happens just during or before menstruation period.^[1,2]^ It has been reported as a most common gynecologic complaint, with a prevalence between 16% and 91% worldwide.^[3]^ Approximately 65% cases occur in USA,^[4]^ 60% in Canada,^[5]^ while 41.7% in China.^[6]^ Although dysmenorrhea is not life-threatening, it can restrict social and daily routine works in young women,^[7]^ decrease quality of life,^[7,8]^ reduce daily learning and educational activities, and reduce attending at school.^[9]^ Moreover, severe dysmenorrhea may lead to miss of 600 million working hours and loss of $2 billion in productivity annually.^[10]^ The first line treatment option for relieving pain in PD is nonsteroidal antiinflammatory medications,^[11]^ however their side effects including gastro-intestinal disorders, kidney or liver injury are problematic.^[12]^ Therefore, more effective and safe therapies are desired by both doctors and patients.

Acupuncture has been widely accepted for treatment of dysmenorrhea in China for thousands of years^[13]^ and has been gradually recognized as a complementary therapy for PD in a Cochrane review^[14]^ and metaanalysis^[15,16]^ in modern society as well. Different acupuncture-related methods are used for relieving dysmenorrhea, including body acupuncture, ear acupuncture, moxibustion, cupping, injection,^[17]^ and acupressure.^[11]^ Sanyinjiao (SP6) acupuncture point, the intersecting point of liver, kidney and spleen channels, is closely related to the uterus.^[18]^ In the clinical practice of acupuncture, SP6 is found to be one of the most commonly used points for PD with satisfied pain-relief effect,^[19–25]^ particularly in patients with cold-dampness stagnation pattern.^[26]^

According to classic Chinese acupuncture theory, applying proper needling technique, particularly the depth, angle, and direction of the needling at an acupoint, is an essential point which may contribute to the results of acupuncture. Our previous study showed that perpendicular deep needling at SP6 to induce strong needling sensation had better pain-relief effect for PD patients compared to perpendicular superficial needling.^[27]^ However, many patients particularly in western countries did not like needling sensation, and their attitude might affect the manipulation of the acupuncturists.^[28]^ Transverse needling has advantage of less pain, and easy to be accepted in large number of patients who are afraid of needling sensation. One study showed that transverse superficial needling at SP6 on PD is better than that of oral administration of indomethacin enteric-coated tablets.^[29]^ We also found^[30]^ that transverse superficial needling at SP6 had similar significant immediate analgesic effect in PD patients as perpendicular deep needling. However, the underlying mechanism of pain-relief effect of transverse needling remains unclear and worthy of further study.

Studies showed that the menstrual pain is closely related to the decreased uterine blood flow,^[31]^ and a high resistance against blood flow in the uterus was found in doppler indices.^[32]^ Our previous study showed that although perpendicular needling at SP6 could immediately relive menstrual pain, it did not show the effects on uterine arterial blood flow in PD patients.^[23]^ Another animal study revealed that transverse needling had analgesia effect through decreasing prostaglandin (PG) F2α, increasing β-endorphin, nitric oxide, and substance P to relieve uterine cramps, increase blood flow.^[33]^ Therefore, we hypothesized that compared with perpendicular needling, the analgesia effect of transverse needling at SP6 may be achieved by better improving uterine arterial blood flow.

This study is designed to investigate the immediate analgesic effect and uterine arterial blood flow changes of transverse needling at SP6 versus perpendicular needling for treatment of PD.

## 2 Methods

### 2.1 Design and setting

**Figure 1:**
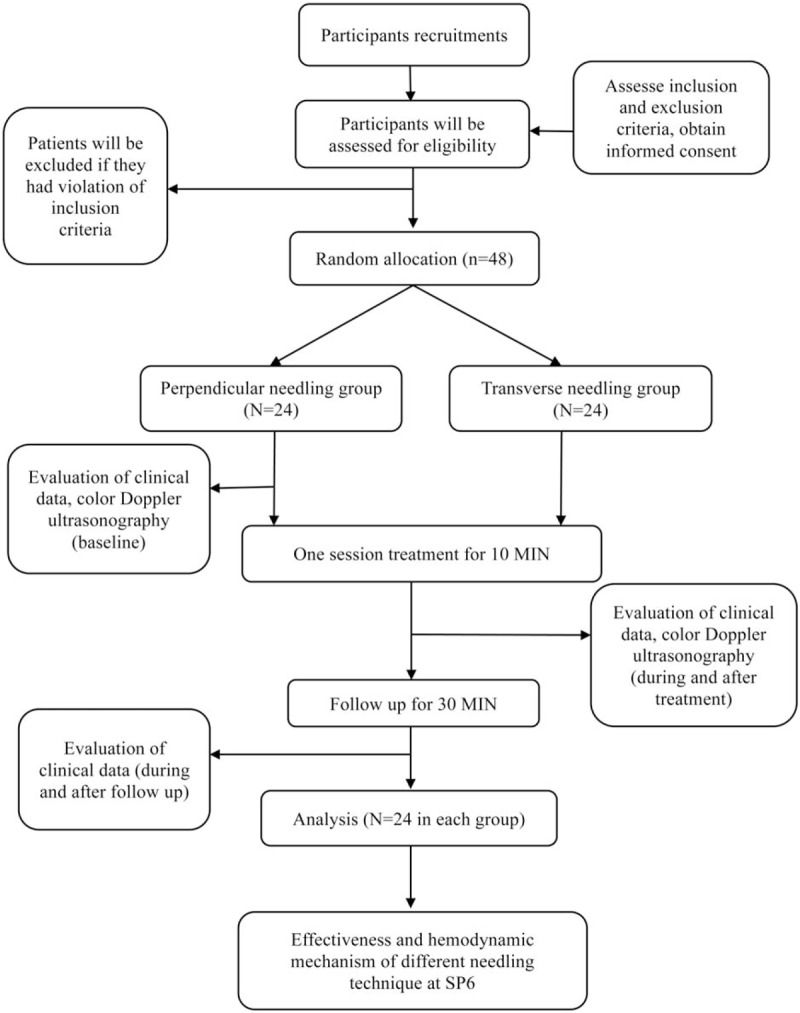
Flowchart of the study.

A prospective randomized, patient-assessor-blinded, controlled trial was designed in Beijing University of Chinese Medicine (BUCM) and will be conducted in 3rd affiliated hospital of BUCM, China from September 30 2019 till July 31 2020. Participants who meet the diagnostic criteria for PD of the consensus guideline,^[11]^ and cold–dampness stagnation pattern according to a revised Chinese national guide line^[34]^ will be randomly allocated in a ratio of 1:1 to either perpendicular needling group or transverse needling group. This study protocol has been approved by the supervision of the ethics committee of the 3rd affiliated hospital of BUCM (ethical approval number BZYSY-2019KYKTPJ-04). This study will be performed according to the guidance and principles of the declaration of Helsinki. The authors retain full control of the manuscript content. The flowchart of this trial is shown in Figure 1.

### 2.2 Participants

Participants will be recruited through multimodal strategies, including posters in the campus of BUCM and nearby 2 universities, and advertisements in social media (WeChat). The posters will contain brief introductions about the protocol of study, inclusion and exclusion criteria and the contact information of the researchers, who will enroll the patients. Every potential participant with interest in the study will contact the trial personnel and undergo a basic evaluation. Those who decide to participate, go through the preliminary evaluation, and provide written informed consent will undergo further examination and ultrasonography at the hospital. Finally, those who satisfy all the inclusion criteria will be enrolled. Participants will return on the first day of their next menstrual cycle for treatment when the pain level was confirmed to be ≥40 mm on the visual analog scale for pain (VAS-P) scale.

#### 2.2.1 Sample size

In this study, the sample size was calculated according to following repeated measurement formula: 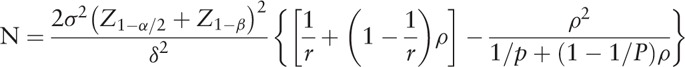


Where N is the sample size of each groups, α is the significance level (0.05), β is related to study power (0.2) σ^2^ is the variance of measurements, δ is minimum effect size, *p* is the number of observations before treatment of each participant, *r* is the number of observations after treatment of each participant and ρ = 0.65. Based on our previous pilot study^[30]^ with the standard deviation of VAS-P values of 19.67 after 30 minute treatment and minimum clinical effective changes of VAS-P of 10 mm, we calculated that a sample size of 20 per group was a necessary to provide 80% power to detect a difference in the VAS-P value between two groups, considering a 20% drop-out rate, 24 participants are required per group.

#### 2.2.2 Inclusion criteria

Participants will be eligible if they

(1) Are aged between 18 to 30 years old, normal menstrual cycle (28 ± 7 days) in the last 6 months,
(2) Have diagnosed as PD,^[11]^
(3) Have also diagnosed as cold-dampness stagnation pattern according to traditional Chinese medicine (TCM),^[34]^
(4) Have scores 40 mm on 100 on the VAS and no oral administration of any analgesic 24 hours before trial, and
(5) Provide written informed consent.

#### 2.2.3 Exclusion criteria

Participants with any of the following conditions will be excluded:

(1) Secondary dysmenorrhea caused by endometriosis, uterine myomas and polyps, pelvic inflammatory disease,
(2) Irregular menstrual cycle, lactation, asthma, severe and uncontrolled psychiatric disorders, life-threatening conditions (eg, sever cardiovascular, liver or kidney diseases),
(3) History of receiving treatment for PD within the last 3 months,
(4) Participants who is familiar or has previous information about acupuncture, and women who have no compliance for acupuncture treatment will also be excluded.

#### 2.2.4 Withdraw criteria

Participants could withdraw from the study due to any reason at any time and will not affect the doctor's treatment. Participants who have developed severe adverse events will also withdraw from the study and will be monitored till its resolution.

### 2.3 Randomization

Participants will be randomized to either the perpendicular needling group or transverse needling group by an independent statistician from BUCM using computer-generated random allocation sequence in SPSS 20.0 software (IBM, Armonk, NY, USA) in ratio of 1:1. The acupuncturist will contact the randomization center by telephone for the group allocation of the participants, who meet the eligibility criteria after signing an informed consent and completing all baseline assessments. This procedure will ensure adequate randomization concealment.

### 2.4 Blinding

In this study, the acupuncturist cannot be blinded to group allocation due to the specific nature of intervention. But, participants, outcome assessors, data collectors and statisticians will be blinded throughout the study.

### 2.5 Interventions

**Figure 2:**
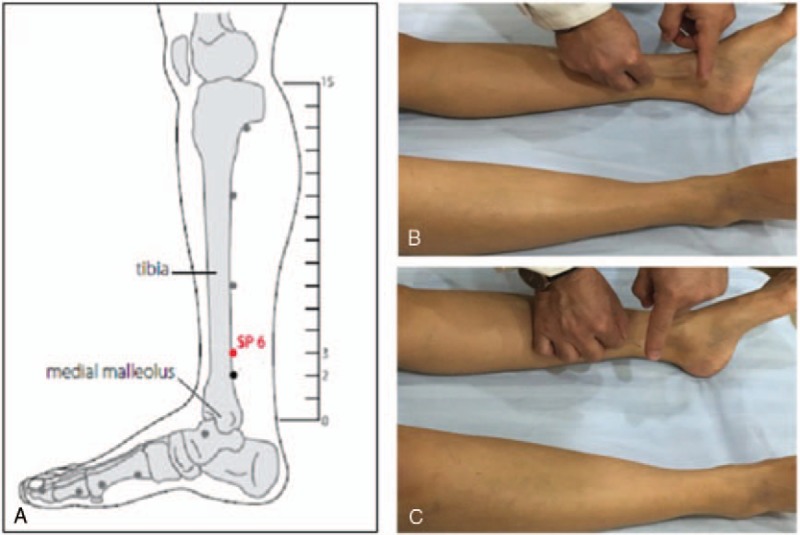
Location of the acupoint and 2 needling techniques. A: Location of the SP6; B: Tube needling at SP6 transversely; C: Tube needling at SP6 perpendicularly. SP6 = Sanyinjiao.

Acupuncture treatment will be implemented, when the participants VAS-P score of menstrual pain in the first day of menstruation is equal to or more than 40 mm. Both groups will be received an acupuncture treatment for 10 minutes at bilateral SP6 acupuncture points, after sterilizing the skin on the areas where the needles will be inserted, by using single-use sterile filiform needles of 0.30 × 40 mm (Zhongyan Taihe, Beijing Zhongyan Taihe Medical Instruments center, Beijing, China). In perpendicular needling group, the needles will be vertically inserted to SP6 bilaterally in a depth of 1 to 1.2 cun and will be manipulated by lifting-thrusting and twirling methods for 30 seconds to achieve proper needling sensation. In transverse needling group, the needles will be inserted to bilateral SP6 transversely 1 to 1.2 cun toward the abdomen without any manipulation to avoid needling sensation. The location of the acupoint and 2 needling techniques are shown in Figure 2.

The acupuncture treatments in both groups will be performed by the same acupuncturist with 10 years of clinical experience throughout the study, the room temperature will be kept 25°C and other influencing factors are prevented to avoid bias.

In this study, patients will be instructed to use Aspirin for pain relief, if VAS pain be more than 80 mm.

### 2.6 Outcome measures

The primary outcome measure is VAS-P, and secondary outcome measures are color doppler ultrasonography, Hamilton anxiety scale (HAM-A), the changes of blood pressure (BP) and heart rate (HR), and adverse events.

#### 2.6.1 Primary outcome measure

The severity of dysmenorrhea will be measured by VAS-P, which is continuous quantitative variable varying from 0 mm (no pain) to100 mm (extreme pain). And it has some documented validity and reliability.^[35,36]^ Participants will be assessed by asking to rate the severity of their pain on a standard 100 mm VAS-P at baseline, 5 minutes after needling, 10 minutes after needling (needle removal), and 10 and 30 minutes after removing the needles.

#### 2.6.2 Secondary outcome measure

##### 2.6.2.1 Color doppler ultrasonography^[37]^

Color doppler ultrasonography will be performed to calculate the uterine artery blood flow changes by measuring pulsatility index (PI), resistance index (RI) values, and ratio of systolic peak and diastolic peak (S/D ratio). Color doppler ultrasonography will be made by transabdominal type-B ultrasonic diagnostic apparatus LOGIQ E9 XDclear 2.0 (General Electric Co.) with convex probe (3–5 MHz). All participants will be examined at 14:00 to 17:00 with 25°C room temperature in the first day of menstruation by the same specialist. It will be performed at baseline, 5 minutes after needling and 10 minutes after needling (needle removal).

##### 2.6.2.2 HAM-A

The changes of the anxiety will be measured by HAM-A, which is 1 of the first rating scales developed to measure the severity of anxiety symptoms and is widely used in both clinical trial and clinics.^[38]^ It consists of 14 items and measures both psychic and somatic anxiety. Severity of each items will be scored using a 5-point scale ranging from 0 (not present) to 4 (severe). Patients will be assessed with this scale before needling (baseline measurement) and 30 minutes after removing the needles.

##### 2.6.2.3 BP and HR

The changes of BP and HR of patients will be measured at baseline, 10 minutes after needling (needle removal) and 30 minutes after removing the needles.

##### 2.6.2.4 Blinding assessment

To test whether the participants are blinded successfully, all participants will be asked to guess which kind of acupuncture they received after the treatment.

##### 2.6.2.5 Adverse events

All adverse reactions that will be reported by patients and researchers in both groups during the treatment will be recorded in the case report form (CRF).

The schedule of procedures and assessments is presented in Table 1.

**Table 1:**
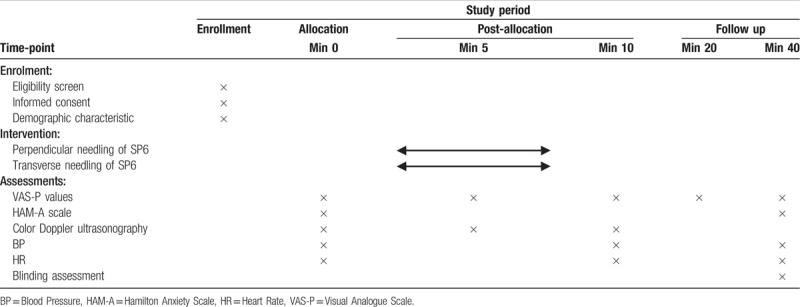
schedule of enrollment, intervention, and assessments.

### 2.7 Data management and quality control

The CRF includes observation time points, interventions, outcome measures, and adverse events. The researchers will be required to follow the requirements of the CRF and fill in the relevant information in a timely and accurate manner. The responsible researcher will supervise and monitor the quality of the study. The recruitment and intervention procedures, and record of the CRF will be checked by regular monitoring.

### 2.8 Statistical analysis

All statistical analyses will be performed using SPSS 20.0 software (IBM, Armonk, NY) by statistician, who is independent from the research team and blinded to patient allocation. The effects of different needling technique on PD will be analyzed on an intention-to-treat (ITT) approach. To evaluate the sensitivity of analysis, the results of the ITT analysis will be compared with those of per-protocol analysis. Baseline demographic and clinical characteristics of participants will be reported as mean (standard deviation) for continuous variables, and as frequencies (percentages) for categorical variables. Repeated measures analysis of variance (ANOVA) will be used for the analysis of variables, which will be measured at several different times. For other variables, 1-way ANOVA or non-parametric Mann-Whitney *U* test will be applied. Mean differences from baseline to post-treatment in each group will be assessed using paired *t* tests or Wilcoxon signed rank tests. *P* < .05 will be considered significant.

## 3 Discussion

This study is designed to illustrate the influence of needling techniques on the therapeutic result of the acupoint. It will perform perpendicular or transverse needling with same length of needle body embedded in a solo acupoint and compare analgesia and hemodynamic responses right after needling.

### 3.1 Needling technique is essential for efficacy of acupoints

The use of specific needling techniques for treatment of different type of pathology can be traced back to Huangdi Neijing.^[39]^ Various needling techniques such as subcutaneous needling, triple-directional needling, waggle needling, and short needling recorded in Neijing are usually used for relevant disorders of skin, muscle, sinew, and bone, respectively. With gradual development in each generation, the perception of applying needling technique which should be suitable for the disease is now widely accepted by modern acupuncturists.

Needling technique, is considered as one of the most significant factors in the curative efficacy of acupoints, which related to depth, intensity and duration.^[40]^ There is both theoretical and empirical support for the hypothesis that alterations in needling technique have an impact on therapeutic outcomes. Sun et al in their systematic review, found relation between needling depth and therapeutic efficacy in lumbar disk herniation.^[41]^ Studies reported that the different depths which were used in acupuncture needling can lead to different impression on the central nervous system and different clinical therapeutic effects.^[42,43]^

According to the clinical experience of acupuncture practice, needling depth and angle of insertion are always considered in combination. Thus, some innovative needling methods such as ankle-wrist acupuncture^[44]^ and Fu's subcutaneous needling^[45]^ are developed, with advantages of no or less needling sensation. As a classic acupoint for PD, the needling techniques at SP6 in the previous studies focused only on needling depths,^[27]^ or only compare transverse needling with medications.^[29]^ At present, there are few attentions paid to the influence of comprehensive factors of needling including depth and angle, on the efficacy of same acupoint. Therefore, the efficacy of transverse subcutaneous needling at SP6 for PD should be investigated further in clinical trials. In addition, because it lacks a clear scientific explanation, the underlying mechanism deserves further study.

In current study, sham acupuncture group is not set. The main reason is that the main purpose of this study is to compare the effective of 2 needling techniques at SP6 for PD, so it is a noninferiority trail.^[46]^ Moreover, those 2 treatment methods have been proven effective in previous studies. For example, perpendicular needling at SP6 showed more effective results for PD compared with other acupoint, nonacupoint and waiting list controls in a series studies of our research group,^[19,22,23]^ particularly in cold-damp stagnation TCM pattern^[26]^ and compared with nondeqi needing (similar to the intervention of sham acupuncture in the majority of studies).^[27]^ Superficial transverse needling at the location of SP6 on PD patients also showed better pain-relief effect than analgesia medication.^[29,47]^ A latest review^[48]^ pointed that placebo might not be as distinctively defined as it is necessary for conducting a clinical trial in the non-pharmacological arena, such as acupuncture, and pragmatic approach should aim to maximize all the positive factors of a treatment that can be recruited while minimizing risk and negative effects.

### 3.2 Combination of subjective and objective measures is necessary for pain assessment

Pain is a complex, multifaceted and subjective experience, which makes a number of measurement challenges. It is important that researchers use sensitive and accurate tools for pain outcome assessments. Currently, there is no valid and reliable method, which objectively quantifies a patient's experience of pain.^[49]^

In the current study, to evaluate subjectively reported symptoms, we will use the VAS-P as the primary outcome measure to assess pain of the patients, and the HAM-A scale also used as secondary outcome. Studies mentioned that women with psychological problems like to have decreased pain thresholds compared to psychologically healthy women. Anxiety and depression affect the patients’ perception of pain, pain behaviors, pain explanation, and also managing response to pain. Therefore, the human's mental health condition could make her susceptible to more severe dysmenorrhea.^[50]^ In addition to subjective assessments by the patients, color doppler ultrasonography will be used to evaluate the uterine artery blood flow changes by measuring PI, RI values, and ratio of S/D ratio. Therefore, we will combine the subjective and objective measures to assess the menstrual pain in the present study.

### 3.3 Uterine blood flow directly reflects the immediate analgesia effect of acupuncture for PD *in vivo*

The mechanisms of acupuncture analgesia remain unclear, particularly for visceral pain.^[51]^ Beside the widely accepted understanding of increased PG release to explain the pathogenesis of PD,^[31]^ more studies focused on investigating uterine blood flow using doppler ultrasonography in human studies. Those studies showed that uterine artery blood flow reduced in dysmenorrhea patients with higher PI and RI values and S/D ratio, leading to resultant myometrial ischemia, and hence pain.^[32,52]^ Moreover, the severity of symptoms in PD could be reflected by doppler index values.^[37]^

In terms of TCM theory, pain is always due to “qi and blood stagnation”. The menstrual pain in PD patients with cold-dampness stagnation pattern is typically caused by impeded blood circulation due to cold-dampness accumulation. Interestingly, another 3-dimensional power doppler assessment of uterine vascularization study found there was significant intrauterine blood flow stasis specifically in women with severe PD,^[53]^ showing similar understanding on the pathogenesis of PD in both modern medicine and TCM. Animal study found that acupuncture at SP6 had immediate analgesia effect in PD rats by improving uterine microcirculation.^[54]^ Moreover, although transverse needling technique has been proven effective for PD,^[29,44]^ there was no study to reveal its mechanism. Therefore, color doppler ultrasonography has been used as a tool with which to reveal the hemodynamic mechanism of immediate analgesia effects of different needling techniques at SP6 for PD in vivo in the current study.

Considering the characteristics of ultrasonography study on blood flow, to ensure the stability of data acquisition, only one technician will perform all ultrasonography using the same machine at the same period of time (between 14:00–17:00 of each measurement) according to the related operation standard and design of the study in the fixed condition.

### 3.4 Advantages

To the best of our knowledge, this trial will be the first study protocol designed to explore the influence of needling techniques on the analgesia effect of solo acupoint and its hemodynamic responses for PD. The strengths in methodology, including ensure the stability of color doppler ultrasonography data acquisition, rigorous randomized, participants-blinded, and assessors-blinded, will guarantee the quality of this study.

We expected that our findings will provide a further explanation of the hemodynamic mechanism by which different needling technique at SP6 for menstrual pain and promote more widespread awareness of the benefits of needling techniques on the effects of acupoints in acupuncture clinical setting.

### 3.5 Limitations

The main limitation of this study is the small sample size. Second, there is only 1 treatment session and the time of needle retention is relative shorter due to the tolerance of the participants, which may reduce the effects of acupuncture. Although the purpose of this trial is to compare the influence of needling techniques on the effect of acupoint, lack of sham acupuncture group as control may reduce power to verify the efficacy of acupuncture at a certain degree.

## Acknowledgments

The author would like to thank to all participants of the study. We especially appreciate all the collaborators in 3rd affiliated hospital of BUCM.

## Author contributions

All authors involved to development of the protocol. MRAF involved in research design, primary writing of study protocol, writing of manuscript and will responsible for assessment of patients. AM involved in research design, primary writing of study protocol and will responsible for acupuncture treatment. LMX conceived the idea of the trial, involved in research design, revised and reviewed the manuscript, will supervise the study and has the final responsibility for the decision to submit for publication. YS, JDM, WYY, JXW, and YYG helped in primary writing of study protocol and will responsible for recruitment and management of patients. YT, XQ, and TYS will responsible for recruitment and management of patients. SI involved in writing and editing manuscript. All of the authors have read and approved the final manuscript.

Liang-xiao Ma orcid: 0000-0001-9851-0225.
